# A novel radiomics model combining GTVp, GTVnd, and clinical data for chemoradiotherapy response prediction in patients with advanced NSCLC

**DOI:** 10.3389/fmed.2025.1596788

**Published:** 2025-07-24

**Authors:** Ya Li, Min Zhang, Yong Hu, Dan Zou, Bo Du, Youlong Mo, Tianchu He, Mingdan Zhao, Benlan Li, Ji Xia, Zhongjun Huang, Fangyang Lu, Bing Lu, Jie Peng

**Affiliations:** ^1^Department of Oncology, The Second Affiliated Hospital of Guizhou Medical University, Kaili, China; ^2^Department of Oncology, Affiliated Hospital of Guizhou Medical University, Guiyang, China; ^3^Department of Oncology, Affiliated Cancer Hospital of Guizhou Medical University, Guiyang, China; ^4^Division of Oncology, School of Clinical Medicine, Guizhou Medical University, Guiyang, China; ^5^Department of Oncology, Guiyang Pulmonary Hospital, Guiyang, China; ^6^Department of Oncology, Qiandongnan Prefecture People's Hospital, Kaili, China; ^7^Department of Oncology, Qiannan Prefecture Hospital of Traditional Chinese Medicine, Duyun, China

**Keywords:** NSCLC, radiomics, chemoradiotherapy, GTVp, GTVnd

## Abstract

**Background:**

Numerous radiomic models have been developed to predict treatment outcomes in patients with NSCLC receiving chemotherapy and radiation therapy. However, computed tomography (CT) radiomic models that integrate the Gross Tumour Volume of the primary lesion (GTVp), the Gross Tumour Volume of nodal disease (GTVnd), and clinical information are relatively scarce and may offer greater predictive accuracy than models focusing on GTVp alone. This study aimed to evaluate the efficacy of a CT radiomic model combining GTVp, GTVnd, and clinical data for predicting treatment response in unresectable stage III–IV NSCLC patients undergoing concurrent chemoradiotherapy.

**Methods:**

A total of 101 patients with unresectable stage III–IV NSCLC were included. GTVp was delineated using lung windows, and GTVnd was delineated using mediastinal windows. Radiological features were extracted using Python 3.6, then subjected to F-test and Lasso regression for feature selection. Logistic regression was performed on the selected radiological features. Clinical information was analysed with univariate and multivariate logistic regression to identify significant clinical variables. Five models were developed and evaluated, incorporating GTVp, GTVnd, and clinical data.

**Results:**

The GTVp-based radiomics model achieved an area under the curve (AUC) of 0.855 in the training cohort and 0.775 in the validation cohort. The multimodal composite model (integrating GTVp, GTVnd, and clinical parameters) significantly outperformed the GTVp-only model, with a training AUC of 0.862 and validation AUC of 0.863, demonstrating superior predictive performance for concurrent chemoradiotherapy response in this patient population.

## 1 Introduction

Lung cancer has a high incidence and mortality rate, with an estimated five-year survival of only around 23% (1). It is classified into non-small cell lung cancer (NSCLC) and small cell lung cancer (SCLC) based on pathological features, with NSCLC accounting for approximately 85% of cases (2). For patients with inoperable stage III–IV NSCLC, concurrent chemoradiotherapy (CCRT) is a vital treatment approach (3). However, treatment sensitivity varies among individuals (4, 5), affecting prognosis. Notably, the response to cancer therapy is closely linked to prognosis. Notably, patients who respond more favourably to therapy often experience longer progression-free and overall survival then those with poorer responses (6–8).

Imaging remains the primary method for tumour evaluation in clinical practice (9), and radiomics has emerged as a non-invasive, effective tool for prognostic prediction (10–14). Several radiological models have been developed to predict treatment response and outcomes in patients with NSCLC undergoing CCRT (15–17). Approximately 60% of patients with NSCLC present with advanced or locally advanced disease at diagnosis (18), often because of late detection of non-specific symptoms (19), which can lead to mediastinal lymph node metastasis. In such cases, radiation oncologists typically delineate the Gross Tumour Volume of the primary lesion (GTVp) and nodal disease (GTVnd) for chest radiation therapy. However, when extracting CT radiomic features, many researchers focus solely on GTVp while overlooking GTVnd (20, 21). This omission is notable because pre- and post-treatment changes in GTVnd are equally critical for tumour staging (22). Moreover, prior research has shown that combining mediastinal window CT images with lung window CT images can improve both the malignancy of a nodule and its potential indolence (23, 24). Thus, incorporating GTVnd CT images may be crucial for assessing CCRT efficacy.

Despite the demand for multimodal biomarkers in NSCLC management, no prior study has simultaneously integrated CT radiomics features of GTVp (lung window) and GTVnd (mediastinal window) with clinical parameters to predict CCRT response. Therefore, this study aims to develop and validate a composite model, specifically evaluating its performance in predicting short-term CCRT efficacy among patients with unresectable stage III-IV NSCLC.

## 2 Methods

The study received approval from the Ethics Committee of the Second Affiliated Hospital of Guizhou Medical University (SAHGMU; approval number 2020-LS-03) and was conducted in strict accordance with the Declaration of Helsinki. Informed consent was obtained from all participants.

**Figure 1:**
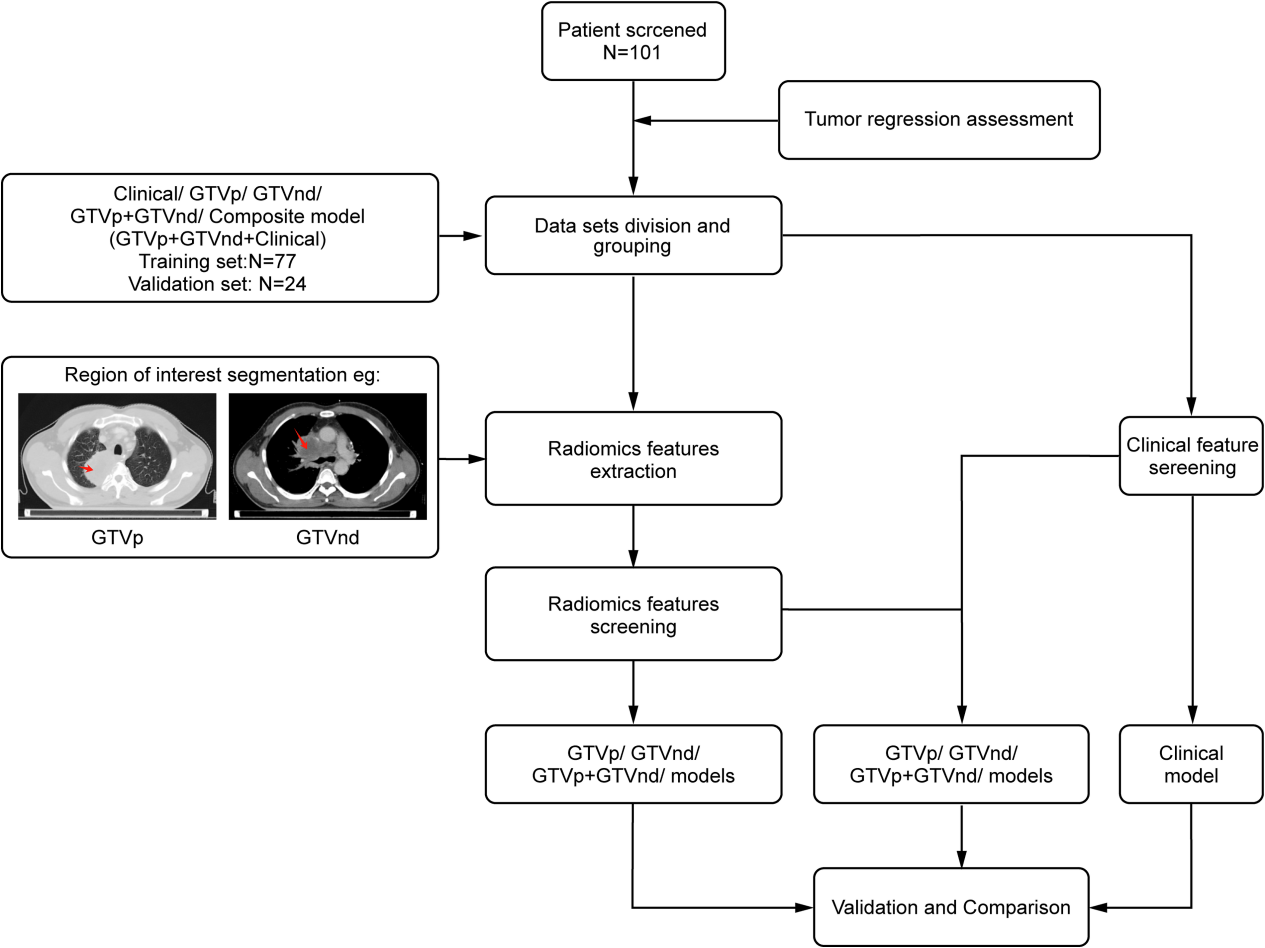
Experimental flowchart.

Figure 1 presents the study flowchart. The inclusion criteria were: (1) pathologically confirmed NSCLC; (2) no surgical indications; (3) no prior therapies (including neoadjuvant chemotherapy, interventional therapy, immunotherapy, or targeted therapy) before CCRT; (4) stage III or IV disease with confirmed mediastinal lymph node metastasis (N2/N3) based on the 8th edition UICC Tumor-Node-Metastasis staging system; (5) availability of standard contrast-enhanced chest CT images obtained within 1 month before and 3 months after treatment completion; and (6) receipt of conventional fractionated radiotherapy (target dose: 60–66 Gy/30–33\u00B0F, intensity-modulated radiotherapy) combined with chemotherapy. For squamous cell carcinoma, weekly paclitaxel plus cisplatin was used, whereas for non-squamous cell carcinoma, pemetrexed was administered every 3 weeks alongside cisplatin (25). The exclusion criteria were: (1) concomitant malignancies, (2) incomplete or poor-quality CT images, and (3) insufficient follow-up data.

This multicentre retrospective study enrolled patients from two distinct cohorts: (1) 77 patients treated at SAHGMU; (2) 24 patients from three regional hospitals (Guiyang Pulmonary Hospital, Qiandongnan People’s Hospital, Qiannan Traditional Chinese Medicine Hospital). All cases were recruited consecutively between January 2019 and July 2023. Treatment outcomes were categorized as complete response (CR), partial response (PR), stable disease (SD), or progressive disease (PD) according to RECIST 1.1 (26). Patients with CR or PR were classified into the treatment-sensitive group, while those with SD and PD were classified as treatment-insensitive.

Chest contrast-enhanced CT images were preprocessed using MATLAB 2014b^1^ with: (1) Spatial normalization: Rigid registration to the INHALE chest CT atlas via ANTs (v2.3.3) using mutual information; (2) Isotropic resampling: Resampling normalized images to 1 mm isotropic voxels using B-spline interpolation. Following the guidelines of ICRU 83 (27), a radiation oncologist with 10 years of experience in lung cancer treatment delineated the GTVp and GTVnd without access to patient information. ITK-SNAP (version 3.8.0; http://www.itksnap.org) was used to manually label slices layer-by-layer (28). GTVp was delineated in the lung window (WW 1600 HU, WL − 600 HU), and GTVnd in the mediastinal window (WW 250 HU, WL 50 HU). The criteria for defining GTVnd included: (1) short-axis diameter ≥1 cm, (2) presence of ≥3 clustered lymph nodes within a single station, (3) pathological confirmation of metastasis in mediastinal lymph nodes (in select patients), or (4) PET-CT SUVₘₐₓ > 2.5 in the region (in select patients). After completing the annotations were completed, the region of interest (ROI) was designated. For each patient, 1,834 radiological features were extracted from the ROIs. These features were standardized using the Z-score and then screened by an F-test in ANOVA, where F is defined as the ratio of between-group variance to within-group variance. To avoid overfitting, LASSO regression with 10-fold cross-validation (via glmnet in R) was performed on each training subset to select the *λ* minimizing mean square error. Only features selected in ≥80% of folds were retained for the final model. Finally, logistic regression was used to construct the radiological models.

Clinical data—including sex, ethnicity, age, smoking history, pathological type, tumour stage, and haematological markers measured 1 week before treatment (such as carcinoembryonic antigen, neuron-specific enolase [NSE], white blood cell count, haemoglobin, and platelet levels)—were collected and initially analysed via univariate regression. Factors with *p* < 0.05 underwent multivariate regression, and variables remaining significant (*p* < 0.05) were incorporated into a clinical prediction model built through logistic regression. PyRadiomics was used for radiomic feature extraction (v3.0.1; https://github.com/radiomics/pyradiomics) (29). Statistical modeling was conducted in R (v3.5.1; https://www.r-project.org/). SPSS (v26.0, IBM Corp., Armonk, NY, USA) handled descriptive statistics.

Combination models were constructed using logistic regression with selected radiological and clinical features. Model performance was evaluated through Receiver Operating Characteristic (ROC) curves, Area Under the Curve (AUC), accuracy, precision, recall, and Decision Curve Analysis (DCA). Statistical significance was defined as *p* < 0.05 for all hypothesis tests.

## 3 Results

A total of 101 participants met the inclusion criteria. Patients were recruited from the SAHGMU (*n* = 77), Guiyang Pulmonary Hospital (*n* = 13), Qiandongnan Prefecture People’s Hospital (*n* = 5), Qiannan Prefecture Traditional Chinese Medicine Hospital (*n* = 6). Table 1 shows the clinical information. Guizhou—an ethnically diverse province in southwest China—is home to all four treatment centres included in this study. The principal ethnic groups were Han (39.60%), Miao (29.70%), and Dong (25.74%). The training cohort and external validation cohort exhibited comparable treatment efficacy rates (*p* > 0.05). Table 2 presents the relationship between clinical features and CCRT treatment sensitivity. After screening, only haemoglobin was significantly correlated with CCRT treatment sensitivity. However, as shown in Table 3, the haemoglobin-based clinical model underperformed among the models, with an AUC of 60.65% in the training set and 65.00% in the validation set.

**Table 1:** Baseline characteristics of patients.

Variables
Categories	Total (*n* = 101)	Training (*n* = 77)	External validation (*n* = 24)	*P*
Sex, *n* (%)	Female	17 (16.83)	15 (19.48)	2 (8.33)	0.583
	Male	84 (83.17)	62 (80.52)	22 (91.67)	
Age, *n* (%)	≤50	17 (16.83)	15 (19.48)	2 (8.33)	0.336
	>50	84 (83.17)	62 (80.52)	22 (91.67)	
Ethnicity, *n* (%)	Miao	30 (29.70)	27 (35.06)	3 (12.50)	0.005
	Dong	26 (25.74)	22 (28.57)	4 (16.67)	
	Han	40 (39.60)	23 (29.87)	17 (70.83)	
	Others	5 (4.95)	5 (6.49)	0 (0.00)	
Efficacy, *n* (%)	CR/PR	28 (27.72)	24 (31.17)	4 (16.67)	0.166
	SD/PD	73 (72.28)	53 (68.83)	20 (83.33)	
Histology, *n* (%)	LUSC	65 (64.36)	46 (59.74)	19 (79.17)	0.238
	LUAD	31 (30.69)	26 (33.77)	5 (20.83)	
	Other	5 (4.95)	5 (6.49)	0 (0.00)	
TNM, *n* (%)	III	67 (66.34)	53 (68.83)	14 (58.33)	0.342
	IV	34 (33.66)	24 (31.17)	10 (41.67)	
CEA, *n* (%)	Normal	59 (58.42)	45 (58.44)	14 (58.33)	0.993
	Elevated	42 (41.58)	32 (41.56)	10 (41.67)	
NSE, *n* (%)	Normal	72 (71.29)	55 (71.43)	17 (70.83)	0.955
	Elevated	29 (28.71)	22 (28.57)	7 (29.17)	
WBC, *n* (%)	Reduced	3 (2.97)	3 (3.90)	0 (0.00)	0.548
	Normal	86 (85.15)	66 (85.71)	20 (83.33)	
	Elevated	12 (11.88)	8 (10.39)	4 (16.67)	
Hb, *n* (%)	Reduced	71 (70.30)	53 (68.83)	18 (75.00)	0.564
	Normal	30 (29.70)	24 (31.17)	6 (25.00)	
PLT, *n* (%)	Reduced	5 (4.95)	3 (3.90)	2 (8.33)	0.567
	Normal	89 (88.12)	69 (89.61)	20 (83.33)	
	Elevated	7 (6.93)	5 (6.49)	2 (8.33)	

LUSC, Lung squamous cell carcinoma; LUAD, Lung adenocarcinoma; CEA, carcinoembryonic antigen; NSE, neuron specific enolase; WBC, White blood cell; Hb, Hemoglobin; PLT, Platelet; Alb, Albumin.

**Table 2:** Clinical model: clinical features related to CCRT sensitivity.

Variables	Univariate analysis		Multivariate analysis	
	OR (95%CI) *P*		OR (95%CI) *P*	
Sex				
Female	1.00 (Reference)			
Male	0.77 (0.23 ~ 2.60)	0.673		
Age (years)				
≤50	1.00 (Reference)			
>50	0.13 (0.02 ~ 1.05)	0.055		
Ethnicity				
Miao	1.00 (Reference)			
Dong	2.10 (0.61 ~ 7.23)	0.239		
Han	1.17 (0.42 ~ 3.22)	0.766		
Others	2.00 (0.20 ~ 20.33)	0.558		
Histology				
LUSC	1.00 (Reference)			
LUAD	0.59 (0.23 ~ 1.50)	0.270		
Other	1.31 (0.14 ~ 12.55)	0.817		
TNM				
III	1.00 (Reference)			
IV	3.03 (1.04 ~ 8.88)	0.043		
CEA				
Normal	1.00 (Reference)			
Elevated	1.40 (0.57 ~ 3.46)	0.459		
NSE				
Normal	1.00 (Reference)			
Elevated	0.51 (0.20 ~ 1.28)	0.149		
WBC				
Reduced	1.00 (Reference)			
Normal	0.00 (0.00 ~ Inf)	0.991		
Elevated	0.00 (0.00 ~ Inf)	0.991		
Hb				
Normal	1.00 (Reference)		1.00 (Reference)	
Reduced	2.85 (1.14 ~ 7.16)	0.025	2.85 (1.14 ~ 7.16)	0.025
PLT				
Reduced	1.00 (Reference)			
Normal	4.57 (0.72 ~ 29.14)	0.108		
Elevated	2.00 (0.19 ~ 20.61)	0.560		

OR: Odds Ratio, CI: Confidence Interval; LUSC, Lung squamous cell carcinoma; LUAD, Lung adenocarcinoma; CEA, carcinoembryonic antigen; NSE, neuron specific enolase; WBC, White blood cell; Hb, Hemoglobin; PLT, Platelet.

**Table 3:** Performance of the models.

Model	Accuracy	Precision	Recall	F1-score	AUC
Clinical					
Training set	68.83%	68.83%	100.00%	81.54%	60.65%
Validation set	83.33%	83.33%	100.00%	90.91%	65.00%
GTVp					
Training set	83.12%	85.71%	90.57%	88.07%	85.53%
Validation set	79.17%	82.61%	95.00%	88.37%	77.50%
GTVnd					
Training set	79.22%	80.33%	92.45%	85.96%	73.43%
Validation set	83.33%	86.36%	85.00%	90.48%	37.50%
GTVp + GTVnd					
Training set	83.12%	84.48%	92.45%	88.29%	85.30%
Validation set	83.33%	83.33%	100.00%	90.91%	80.00%
Composite model (GTVp + GTVnd + clinical)					
Training set	83.12%	84.48%	92.45%	88.29%	86.16%
Validation set	83.33%	83.33%	100.00%	90.91%	86.25%

Data in parentheses are 95% CIs. AUC, area under the curve; GTVp, gross tumor volume of the primary lesion; GTVnd, gross tumor volume of nodal disease.

**Figure 2:**
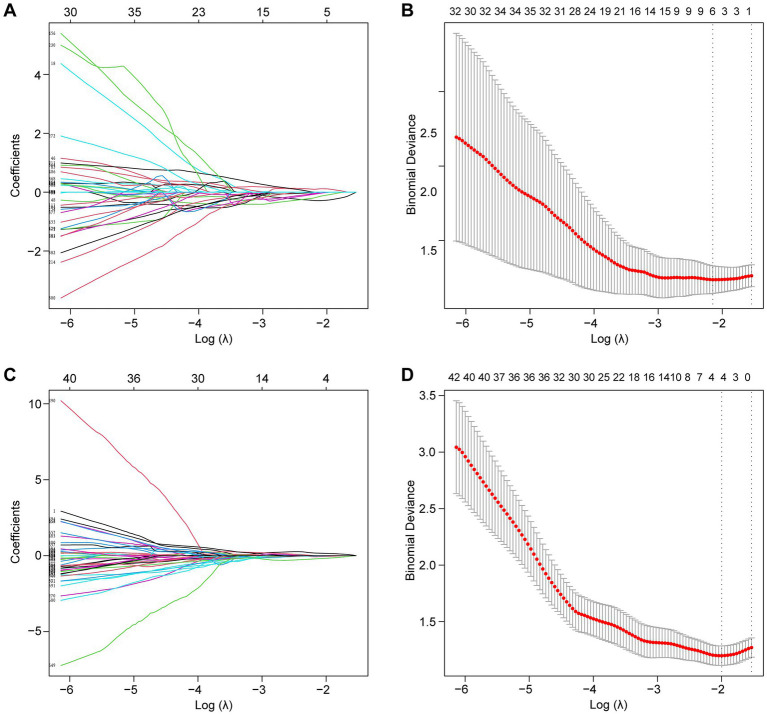
Lasso regression was employed to screen radiological features. **(A)** LASSO coefficient curve for the GTVp group. **(B)** Cross-validation curve for the GTVp group. **(C)** LASSO coefficient curve for the GTVnd group. **(D)** Cross-validation curve for the GTVnd group.

**Figure 3:**
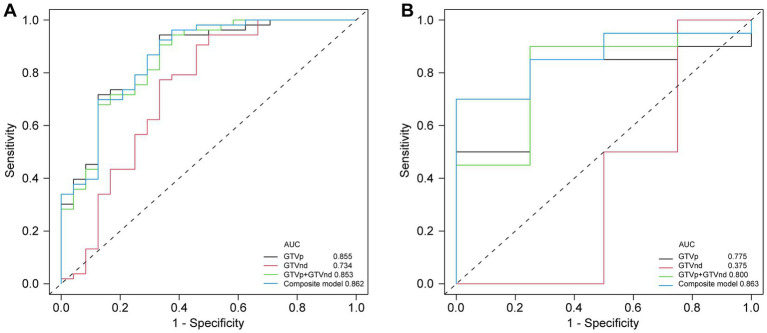
Comparison of ROC curves for different models. **(A)** ROC curves of different radiomic models in the training cohort. **(B)** ROC curves of different radiomic models in the validation cohorts.

Following the F-test and Lasso regression feature selection, six radiomic features were selected for GTVp (lung window) and four for GTVnd (mediastinal window). Figure 2 and Table 4 illustrate the distribution of these selected features. The predictive performance of the radiological models is shown in Figure 3 and Table 3. In the training set, the composite model—incorporating GTVp, GTVnd, and clinical features—achieved the highest AUC (0.862). The second-ranked model was the GTVp-only model (AUC: 0.855), followed by the GTVp + GTVnd combination (AUC: 0.853). The GTVnd-only model yielded the lowest performance (AUC: 0.734). In the external validation set, the composite model again demonstrated the highest accuracy (AUC: 0.863). The GTVp + GTVnd combination ranked second (AUC: 0.800), the GTVp-only model placed third (AUC: 0.775), and the GTVnd-only model performed poorest (AUC: 0.375).

**Table 4:** Selected radiological features.

GTVp	GTVnd
lbp.3D.k_glszm_GrayLevelNonUniformityNormalized	wavelet. LHL_firstorder_10Percentile
lbp.3D.k_glrlm_RunLengthNonUniformityNormalized	wavelet. LHL_glcm_Contrast
original_shape_Sphericity	wavelet. HLH_glszm_SizeZoneNonUniformityNormalized
square_glcm_Imc2	wavelet. LLL_firstorder_InterquartileRange
squareroot_glcm_Correlation	
exponential_glrlm_RunLengthNonUniformity	

**Figure 4:**
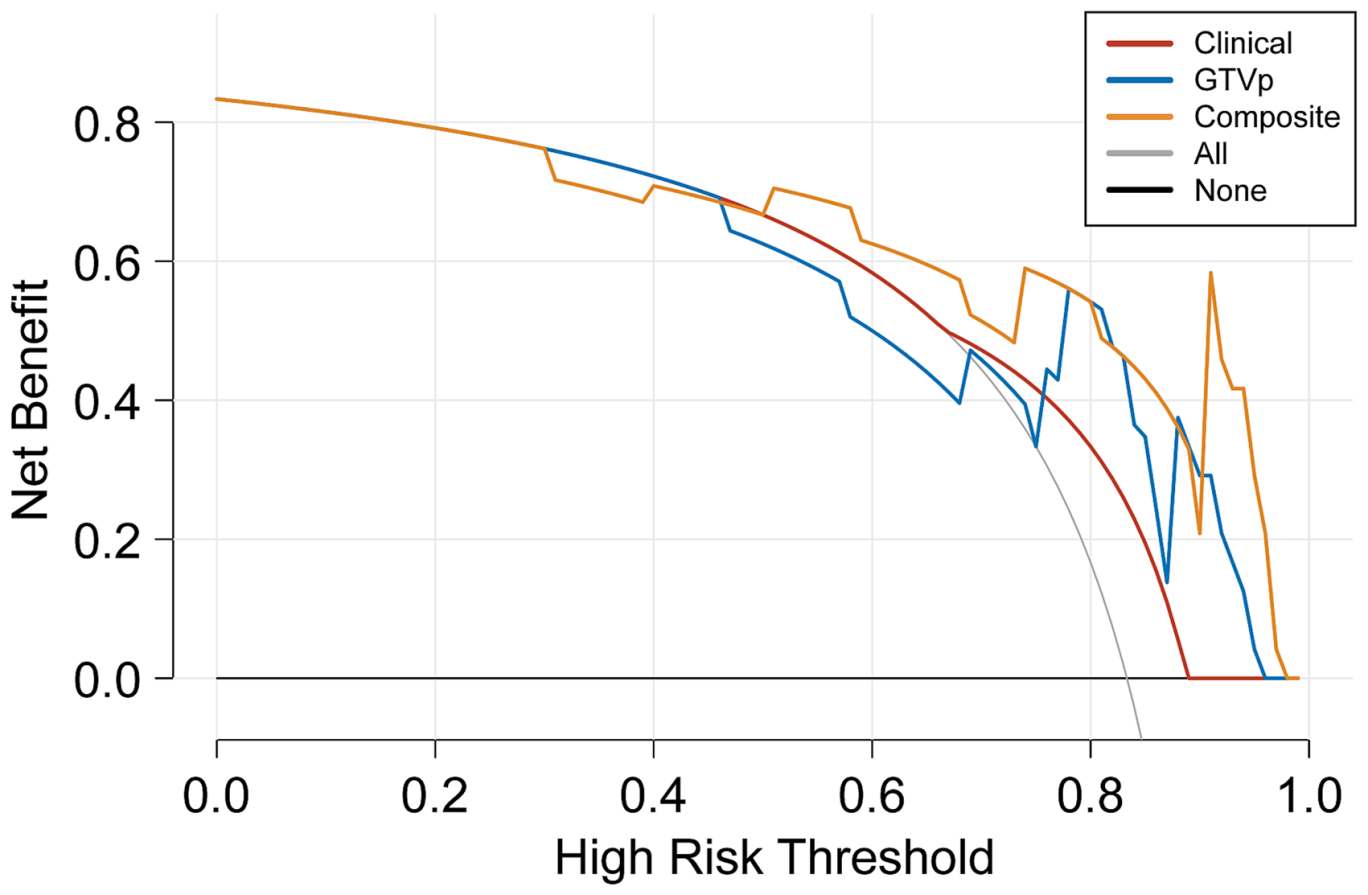
Decision curve analysis of the models.

The DeLong test on the external validation set ROC data (Table 5) showed no statistically significant difference between the composite model and the conventional GTVp model (*p* = 0.14). Considering the limited sample size of the validation cohort (*n* = 24), we conducted clinical decision curve analysis to evaluate real-world utility. As shown in Figure 4, the composite model provided a superior net benefit across threshold probabilities compared to both the conventional clinical model and the GTVp model.

**Table 5:** DeLong test for AUC values of the validation set.

Model	Clinical	GTVp	GTVnd	GTVp+GTVnd	Composite model
Clinical	1	0.59	0.04	0.55	0.26
GTVp	0.59	1	0.23	0.57	0.14
GTVnd	0.04	0.23	1	0.23	0.09
GTVp + GTVnd	0.55	0.57	0.23	1	0.40
Composite model	0.26	0.14	0.09	0.40	1

GTVp, gross tumor volume of the primary lesion; GTVnd, gross tumor volume of nodal disease.

## 4 Discussion

In this study, our radiomic models outperformed the clinical factor model in predicting treatment outcomes. At present, the most commonly used guideline for tumour evaluation is RECIST 1.1; however, metabolic changes in tumour cells induced by chemotherapy and radiation therapy may become apparent earlier than morphological changes (30, 31). While radiation and chemotherapeutic agents effectively inhibit tumour cell proliferation, their structural impact can manifest slowly, making it difficult to detect short-term treatment effects through conventional imaging. Unlike RECIST 1.1, radiomics extracts pre-treatment data from the tumour, thus enabling an earlier assessment of treatment sensitivity before therapy is complete.

Among the 101 patients analysed, decreased haemoglobin emerged as the only clinical feature associated with CCRT sensitivity. Haemoglobin is critical for oxygen transport to tissues. When haemoglobin levels are low, increased anoxia in tumour cells leads to reduced sensitivity to radiotherapy and chemotherapy, ultimately weakening the therapeutic effect (32). In our patient population, over 70% presented with low haemoglobin levels prior to treatment. This could be explained by several factors. First, dietary habits among middle-aged and elderly individuals in Guizhou, who tend to eat more vegetables than meat, can result in insufficient iron intake and anaemia. Second, compromised immunity in cancer patients elevates their risk of secondary infections, which may lead to the excessive destruction of red blood cells. Third, acute and chronic bleeding (e.g., haemoptysis) often associated with lung cancer can further exacerbate anaemia in these patients.

Although the validation set showed that the GTVnd radiomics model alone had a relatively poor predictive performance (AUC: 0.375) compared to the GTVp model (AUC: 0.775), these findings indicate that, in standard CT-based radiomics models for stage III - IV lung cancer, primary tumour features may be more influential than those of metastatic mediastinal lymph nodes. In our study, the radiological features of metastatic mediastinal lymph node lesions sensitive to CCRT all originated from “wavelets” a phenomenon that warrants further inquiry. Moreover, the absence of comprehensive PET/CT scans or mediastinal lymph node biopsies in some patients may have limited the precision of GTVnd delineation, as radiation oncologists relied solely on conventional imaging criteria (e.g., short diameter ≥1 cm or at least three clustered lymph nodes in one region), potentially resulting in a reduced diagnostic rate for positive mediastinal lymph nodes.

We also noted that integrating clinical features with radiological data led to superior predictive performance compared to radiological models alone. The radiomics model combining GTVp and GTVnd (AUC: 0.800) outperformed the individual GTVp and GTVnd models. We compared our model not only to our own previous models but also to similar studies, such as: 1. A 2022 study that used a radiomics nomogram based solely on CT-derived GTVp and clinical features to predict chemoradiotherapy efficacy in locally advanced non-small cell lung cancer, with a training set C-index of 0.796 and a validation set C-index of 0.756 (17); 2. A 2023 study developed a radiomics model based on CT-derived GTVp to predict concurrent chemoradiotherapy in patients with locally advanced non-small cell lung cancer. The study reported that the AUC for the GTV reduction (Criteria A) model was 0.767, while the AUC for the RECIST 1.1 standard (Criteria B) model was 0.771 (16). In contrast, our composite model (GTVp + GTVnd + clinical characteristics) achieved higher AUCs in both the training set (0.862) and the validation set (0.863). Further analysis revealed that the GTVnd features added critical information: (1) “wavelet. LHL_firstorder_10Percentile” quantifies low-intensity pixels in regions with vertical textural detail; (2) “wavelet. LHL_glcm_Contrast” captures roughness/heterogeneity of vertical textures and sensitivity to directional structures; (3) “wavelet. HLH_glszm_SizeZoneNonUniformityNormalized” indicates lesion size heterogeneity; (4) “wavelet. LLL_firstorder_InterquartileRange” stably quantifies slow-varying grayscale distribution in anatomical structures. The inclusion of these GTVnd radiomic features enhanced the model’s efficacy.

In conclusion, our composite model (AUC = 0.863) demonstrated notably better performance than the conventional GTVp model (AUC = 0.775), indicating that including GTVnd radiological features can significantly improve the predictive capacity of CT-based models for CCRT outcomes. Decision curve analysis further confirmed that the composite model provided higher accuracy than the GTVp model alone, highlighting the importance of incorporating additional radiomic features and clinical data in treatment response predictions. This study is the first to show that CT-based radiomic models integrating GTVnd, GTVp, and clinical information can meaningfully enhance CCRT response prediction in unresectable stage III–IV NSCLC. By extracting a broader range of radiomic features, the composite model offers a more comprehensive assessment of the tumour’s biological characteristics, potentially facilitating more individualized cancer treatment strategies. Overall, our findings emphasize the importance of including GTVnd in CT imaging analyses, reinforcing the need for a holistic approach to tumour evaluation.

Despite these promising results, our study has several limitations. First, the use of various CT scanners across four different institutions may have introduced variability in imaging parameters. To reduce this effect, all CT scans were normalized and reconstructed into 1-mm slices. Second, not all patients underwent PET/CT or mediastinal lymph node biopsies, potentially impacting the precision of GTVnd delineation. Previous research indicates that PET/CT is more accurate than conventional CT for detecting malignant lymph nodes (33, 34). Consequently, future research should incorporate PET/CT or biopsy before CCRT to better define GTVnd and improve model accuracy. Third, a single radiation oncologist performed all ROI delineations, restricting our ability to assess inter-observer consistency in radiomic feature extraction. Fourth, due to a relatively small sample size, larger studies are necessary to validate these findings.

## 5 Conclusion

This study demonstrates that a CT-based model integrating GTVp, GTVnd, and clinical data surpasses the conventional GTVp radiological model in predicting CCRT efficacy for patients with unresectable stage III–IV NSCLC. Such an approach may allow for earlier adjustments to treatment regimens for patients expected to have less favourable outcomes.

## Data Availability

The raw data supporting the conclusions of this article will be made available by the authors, without undue reservation.

## Footnotes

^1^ https://ww2.mathworks.cn/

## References

[1] Rebecca LS Kimberly DM Nikita Sandeep W Ahmedin J. Cancer statistics, 2023. CA Cancer J Clin. (2023) 73:17–48. doi: 10.3322/caac.21763 36633525

[2] Molina JR Yang PG Cassivi SD Schild SE Adjei AA. Non-small cell lung cancer: epidemiology, risk factors, treatment, and survivorship. Mayo Clin Proc. (2008) 83:584–94. doi: 10.1016/S0025-6196(11)60735-0, PMID: 18452692 PMC2718421

[3] Maconachie R Mercer T Navani N McVeigh G. Lung cancer: diagnosis and management: summary of updated NICE guidance. BMJ-Brit Med J. (2019) 364:l1049. doi: 10.1136/bmj.l1049 30923038

[4] Zhivotovsky B Joseph B Orrenius S. Tumor Radiosensitivity and apoptosis. Exp Cell Res. (1999) 248:10–7. doi: 10.1006/excr.1999.4452, PMID: 10094808

[5] Zheng WY Xin Y Siyang W Xiaonan W Qiutao W Wenhao C. Instability mechanism of Osimertinib in plasma and a solving strategy in the pharmacokinetics study. Front Pharmacol. (2022) 13:928983. doi: 10.3389/fphar.2022.928983 35935836 PMC9354582

[6] Park C Chu HH Kim JH Kim SY Alrashidi I Gwon DI. Clinical significance of the initial and best responses after chemoembolization in the treatment of intermediate-stage hepatocellular carcinoma with preserved liver function. J Vasc Interv Radiol. (2020) 31:1998. doi: 10.1016/j.jvir.2020.04.017, PMID: 32988715

[7] Pointer KB Katipally RR Bestvina CM Juloori A Partouche J Patel JD. Evaluation of initial metastatic tumor location and radiation response to determine outcomes in patients who received combination stereotactic body radiotherapy and immunotherapy for NSCLC. Int J Radiat Oncol Biol Phys. (2021) 111:e449. doi: 10.1016/j.ijrobp.2021.07.1266

[8] Tsurugai Y Kozuka T Ishizuka N Oguchi M. Relationship between the consolidation to maximum tumor diameter ratio and outcomes following stereotactic body radiotherapy for stage I non-small-cell lung cancer. Lung Cancer. (2016) 92:47–52. doi: 10.1016/j.lungcan.2015.12.003, PMID: 26775596

[9] Kurland BF Gerstner ER Mountz JM Schwartz LH Ryan CW Graham MM. Promise and pitfalls of quantitative imaging in oncology clinical trials. Magn Reson Imaging. (2012) 30:1301–12. doi: 10.1016/j.mri.2012.06.009, PMID: 22898682 PMC3466405

[10] Peng J Kang S Ning Z Deng H Shen J Xu Y. Residual convolutional neural network for predicting response of transarterial chemoembolization in hepatocellular carcinoma from CT imaging. Eur Radiol. (2020) 30:413–24. doi: 10.1007/s00330-019-06318-1, PMID: 31332558 PMC6890698

[11] Jiang X Zhao H Saldanha OL Nebelung S Kuhl C Amygdalos I. An MRI deep learning model predicts outcome in rectal Cancer. Radiology. (2023) 307:e222223. doi: 10.1148/radiol.222223, PMID: 37278629

[12] Peng J Huang J Huang G Zhang J. Predicting the initial treatment response to Transarterial chemoembolization in intermediate-stage hepatocellular carcinoma by the integration of Radiomics and deep learning. Front Oncol. (2021) 11:730282. doi: 10.3389/fonc.2021.730282, PMID: 34745952 PMC8566880

[13] Peng J Lu F Huang J Zhang J Gong W Hu Y. Development and validation of a pyradiomics signature to predict initial treatment response and prognosis during transarterial chemoembolization in hepatocellular carcinoma. Front Oncol. (2022) 12:853254. doi: 10.3389/fonc.2022.853254, PMID: 36324581 PMC9618693

[14] Bera K Braman N Gupta A Velcheti V Madabhushi A. Predicting cancer outcomes with radiomics and artificial intelligence in radiology. Nat Rev Clin Oncol. (2022) 19:132–46. doi: 10.1038/s41571-021-00560-7, PMID: 34663898 PMC9034765

[15] Chen W Wang L Hou Y Li L Chang L Li Y. Combined Radiomics-clinical model to predict radiotherapy response in inoperable stage III and IV non-small-cell lung Cancer. Technol Cancer Res Treat. (2022) 21:15330338221142400. doi: 10.1177/15330338221142400, PMID: 36476110 PMC9742722

[16] Zhou C Hou L Tang X Liu C Meng Y Jia H. CT-based radiomics nomogram may predict who can benefit from adaptive radiotherapy in patients with local advanced-NSCLC patients. Radiother Oncol. (2023) 183:109637. doi: 10.1016/j.radonc.2023.109637, PMID: 36963440

[17] Chen X Tong X Qiu Q Sun F Yin Y Gong G. Radiomics nomogram for predicting Locoregional failure in locally advanced non-small cell lung Cancer treated with definitive Chemoradiotherapy. Acad Radiol. (2022) 29:S53–61. doi: 10.1016/j.acra.2020.11.018, PMID: 33308945

[18] Meza R Meernik C Jeon J Cote ML. Lung cancer incidence trends by gender, race and histology in the United States, 1973-2010. PLoS One. (2015) 10:e0121323. doi: 10.1371/journal.pone.0121323, PMID: 25822850 PMC4379166

[19] Wilk AM Kozłowska E Borys D D'Amico A Fujarewicz K Gorczewska I. Radiomic signature accurately predicts the risk of metastatic dissemination in late-stage non-small cell lung cancer. Transl Lung Cancer Res. (2023) 12:1372–83. doi: 10.21037/tlcr-23-60, PMID: 37577306 PMC10413035

[20] Chen W Hou X Hu Y Huang G Ye X Nie S. A deep learning- and CT image-based prognostic model for the prediction of survival in non-small cell lung cancer. Med Phys. (2021) 48:7946–58. doi: 10.1002/mp.15302, PMID: 34661294

[21] Gong J Bao X Wang T Liu J Peng W Shi J. A short-term follow-up CT based radiomics approach to predict response to immunotherapy in advanced non-small-cell lung cancer. Onco Targets Ther. (2022) 11:2028962. doi: 10.1080/2162402X.2022.2028962, PMID: 35096486 PMC8794258

[22] Asamura H Chansky K Crowley J Goldstraw P Rusch VW Vansteenkiste JF. The International Association for the Study of Lung Cancer lung Cancer staging project: proposals for the revision of the N descriptors in the forthcoming 8th edition of the TNM classification for lung Cancer. J Thoracic Oncol. (2015) 10:1675–84. doi: 10.1097/JTO.0000000000000678, PMID: 26709477

[23] Nasir M Farid MS Suhail Z Khan MH. Optimal thresholding for multi-window computed tomography (CT) to predict lung cancer. Appl Sci. (2023) 13:256. doi: 10.3390/app13127256

[24] Lu H Mu W Balagurunathan Y Qi J Abdalah MA Garcia AL. Multi-window CT based Radiomic signatures in differentiating indolent versus aggressive lung cancers in the National Lung Screening Trial: a retrospective study. Cancer Imaging. (2019) 19:45. doi: 10.1186/s40644-019-0232-6, PMID: 31253194 PMC6599273

[25] Ettinger DS Wood DE Aisner DL Akerley W Bauman JR Bharat A. NCCN guidelines® insights: non-small cell lung Cancer, version 2.2023. J Natl Comprehens Cancer Network. (2023) 21:340–50. doi: 10.6004/jnccn.2023.0020, PMID: 37015337

[26] Eisenhauer EA Therasse P Bogaerts J Schwartz LH Sargent D Ford R. New response evaluation criteria in solid tumours: revised RECIST guideline (version 1.1). Europ J Cancer. (2009) 45:228–47. doi: 10.1016/j.ejca.2008.10.026 19097774

[27] Grégoire V Mackie TR. State of the art on dose prescription, reporting and recording in intensity-modulated radiation therapy (ICRU report no. 83). Cancer Radiother. (2011) 15:555–9. doi: 10.1016/j.canrad.2011.04.003, PMID: 21802333

[28] Yushkevich PA Piven J Hazlett HC Smith RG Ho S Gee JC. User-guided 3D active contour segmentation of anatomical structures: significantly improved efficiency and reliability. NeuroImage. (2006) 31:1116–28. doi: 10.1016/j.neuroimage.2006.01.015, PMID: 16545965

[29] van Griethuysen JJM Fedorov A Parmar C Hosny A Aucoin N Narayan V. Computational Radiomics system to decode the radiographic phenotype. Cancer Res. (2017) 77:e104–7. doi: 10.1158/0008-5472.CAN-17-0339, PMID: 29092951 PMC5672828

[30] Yang Y Tian W Su L Li P Gong X Shi L. Tumor-infiltrating cytotoxic T cells and tumor-associated macrophages correlate with the outcomes of neoadjuvant Chemoradiotherapy for locally advanced rectal Cancer. Front Oncol. (2021) 11:743540. doi: 10.3389/fonc.2021.743540, PMID: 34733785 PMC8560008

[31] Wang HM Wu MH Chang PH Lin HC Liao CD Wu SM. The change in circulating tumor cells before and during concurrent chemoradiotherapy is associated with survival in patients with locally advanced head and neck cancer. Head Neck. (2019) 41:2676–87. doi: 10.1002/hed.25744, PMID: 30903634

[32] Topkan E Selek U Ozdemir Y Yildirim BA Guler OC Mertsoylu H. Chemoradiotherapy-induced hemoglobin nadir values and survival in patients with stage III non-small cell lung cancer. Lung Cancer. (2018) 121:30–6. doi: 10.1016/j.lungcan.2018.04.016, PMID: 29858023

[33] Ventura E Islam T Gee MS Mahmood U Braschi M Harisinghani MG. Detection of nodal metastatic disease in patients with non-small cell lung cancer: comparison of positron emission tomography (PET), contrast-enhanced computed tomography (CT), and combined PET-CT. Clin Imaging. (2010) 34:20–8. doi: 10.1016/j.clinimag.2009.03.012, PMID: 20122515

[34] Al-Sarraf N Gately K Lucey J Wilson L McGovern E Young V. Lymph node staging by means of positron emission tomography is less accurate in non-small cell lung cancer patients with enlarged lymph nodes: analysis of 1,145 lymph nodes. Lung Cancer. (2008) 60:62–8. doi: 10.1016/j.lungcan.2007.08.036, PMID: 17920724

